# Comparison of the audit method and secondary data sources for assessing health-related characteristics of the built environment in a Brazilian city

**DOI:** 10.1590/0102-311XEN061124

**Published:** 2024-12-20

**Authors:** Cristina Borges Cafruni, Anderson Garcez, Vanessa Backes, Ruth Liane Henn, Fernanda Souza de Bairros, Juvenal Soares Dias-da-Costa, Maria Teresa Anselmo Olinto

**Affiliations:** 1 Universidade Federal do Rio Grande do Sul, Porto Alegre, Brasil.; 2 Programa de Pós-graduação em Ciências da Nutrição, Universidade Federal de Ciências da Saúde de Porto Alegre, Porto Alegre, Brasil.; 3 Universidade do Vale do Rio dos Sinos, São Leopoldo, Brasil.; 4 Programa de Pós-graduação em Saúde Coletiva, Universidade do Vale do Rio dos Sinos, São Leopoldo, Brasil.; 5 Departamento de Saúde Coletiva, Universidade Federal do Rio Grande do Sul, Porto Alegre, Brasil.; 6 Programa de Pós-graduação em Alimentação, Nutrição e Saúde, Universidade Federal do Rio Grande do Sul, Porto Alegre, Brasil.; 7 Programa de Pós-graduação em Ciências Médicas: Endocrinologia, Universidade Federal do Rio Grande do Sul, Porto Alegre, Brasil.

**Keywords:** Built Environment, Neighborhood Characteristics, Urban Health, Methods, Geographic Information Systems, Ambiente Construído, Características da Vizinhança, Saúde Urbana, Métodos, Sistemas de Informação Geográfica, Entorno Construido, Características del Vecindario, Salud Urbana, Métodos, Sistemas de Información Geográfica

## Abstract

This study compared the audit method and secondary data sources for assessing health-related characteristics of the built environment in a Brazilian city. Study sample included 45 census tracts chosen by systematic random sampling, and 36 homes selected from each, from a population-based study. Each neighborhood was delimited by a 400m buffer around the midpoint of the homes. The built environment was assessed for dietary intake and physical activity categorized, respectively, into commercial establishments and services, food retail establishments for home consumption, food retail establishments for immediate consumption, physical activity establishments and public leisure spaces. An audit was conducted in 45 neighborhoods. Secondary data were obtained from lists of commercial establishments and services provided by the city council. Data were analyzed using ArcGIS. Agreement between the audit and secondary data was tested by the intraclass correlation coefficient. Analysis was performed for all neighborhoods and according to neighborhood income tertiles. Results show that agreement between the audit and secondary data was good or excellent for most of the dietary intake and physical activity categories. However, agreement differed according to neighborhood income level across three of the five categories, with lower income neighborhoods presenting the worse agreements. Hence, secondary data should be used with caution to assess health-related characteristics of the built environment in the poorest areas of Brazilian cities.

## Resumo

Este estudo objetivou comparar o método de auditoria e fontes de dados secundários para avaliar características relacionadas à saúde do ambiente construído em um município brasileiro. A amostra derivou de um estudo de base populacional que selecionou 45 setores censitários por amostragem aleatória sistemática; em cada setor, 36 domicílios foram selecionados. Cada vizinhança foi determinado por um *buffer* de 400m em torno do ponto médio das casas. Avaliou-se o ambiente construído para atividade física e consumo alimentar, categorizado em estabelecimentos comerciais e de serviços, estabelecimentos de varejo alimentar para consumo domiciliar, estabelecimentos de varejo alimentar para consumo imediato, estabelecimentos de atividade física e espaços públicos de lazer. Uma auditoria foi realizada em 45 vizinhanças. Os dados secundários foram retirados de listas de estabelecimentos comerciais e serviços prestados pela prefeitura. Os dados foram analisados por meio do ArcGIS. O coeficiente de correlação intraclasse foi utilizado para aferir a concordância entre a auditoria e os dados secundários. A análise foi realizada para todos as vizinhanças e de acordo com os tercis de renda da vizinhança. Os achados mostram que a concordância entre a auditoria e os dados secundários foi boa ou excelente para a maioria das categorias de alimentos e atividade física. No entanto, os resultados também mostram que a concordância diferiu de acordo com os níveis de renda da vizinhança em três das cinco categorias, com concordâncias mais baixas nas vizinhanças de baixa renda (categorias de estabelecimentos de varejo de alimentos e de atividades físicas). Portanto, os dados secundários devem ser usados com cautela para avaliar as características relacionadas à saúde do ambiente construído nas áreas mais pobres das cidades brasileiras.

## Resumen

Este estudio tuvo como objetivo comparar el método de auditoría y fuentes de datos secundarios para evaluar las características relacionadas con la salud del entorno construido en un municipio brasileño. La muestra derivó de un estudio de base poblacional que seleccionó 45 sectores censales mediante muestreo aleatorio sistemático; en cada sector se seleccionaron 36 hogares. Cada barrio estaba determinado por un *buffer* 400m alrededor del punto medio de las casas. Se evaluó el entorno construido para la actividad física y el consumo de alimentos, categorizado en establecimientos comerciales y de servicios, establecimientos minoristas de alimentos para consumo domiciliario, establecimientos minoristas de alimentos para consumo inmediato, establecimientos de actividad física y espacios públicos de ocio. Se realizó una auditoría en 45 barrios. Los datos secundarios se recopilaron de listas de establecimientos comerciales y servicios prestados por el ayuntamiento. Los datos se analizaron utilizando ArcGIS. El coeficiente de correlación intraclase se utilizó para evaluar la concordancia entre la auditoría y los datos secundarios. El análisis se realizó para todos los barrios y según los terciles de ingresos del barrio. Los hallazgos muestran que la concordancia entre la auditoría y los datos secundarios fue buena o excelente para la mayoría de las categorías de alimentos y actividad física. Sin embargo, los resultados también muestran que la concordancia difirió según los niveles de ingresos del vecindario en tres de las cinco categorías, con una concordancia menor en los vecindarios de bajos ingresos (categorías de venta minorista de alimentos y de actividades físicas). Por lo tanto, los datos secundarios deben usarse con precaución para evaluar las características relacionadas con la salud del entorno construido en las zonas más pobres de las ciudades brasileñas.

## Introduction

The ecological perspective underlines individual and environmental factors affecting health, including physical activity and dietary habits ^1^ ^,^ ^2^. Urbanization is linked to population health, thus city planning should consider it to reduce noncommunicable diseases ^3^. The built environment, such as land use, transportation and access to healthy food and leisure spaces, is a crucial component of community health ^2^ ^,^ ^4^. Research interest on the relation between the built environment and physical activity and dietary habits in low- and middle-income countries is growing ^5^ ^,^ ^6^ ^,^ ^7^.

One way to assess the built environment is using secondary data sources collected for other purposes by government or commercial organizations ^8^. In developed countries such sources often include spatial information for the study area, enabling analysis with geographic information systems (GIS). In low- and middle-income countries, however, available GIS data may be outdated or inaccurately reflect areas with non-registered commercial establishments and services ^9^. Another limitation is the temporality of the exposure-outcome relation, as it may not verify the permanence of commercial establishments ^10^.

Despite limitations, secondary data remains a low-cost alternative to other methods for characterizing the built environment. Besides facilitating the analysis of large geographic areas, increasing availability of secondary data from governmental or commercial sources underscores the importance of improving their quality ^11^ ^,^ ^12^. Research has compared them to observational community measures which are considered the gold standard ^8^; however, few studies have compared these methods to evaluate food and physical activity establishments ^13^ ^,^ ^14^ ^,^ ^15^, and none have been conducted in low- and middle-income countries. Moreover, no studies compare the general availability of commercial establishments and services from secondary data and audits, an essential aspect of the walkability index ^9^.

A Brazilian study found low validity for secondary data used to identify fruit and vegetable shops ^16^. Limited research has been conducted in other locations across the country using this data-gathering approach. Additionally, secondary data validity can be affected by socioeconomic status ^8^, particularly in low-income neighborhoods with non-registered commercial establishments ^9^. Thus, the present study compares the audit method and secondary data sources for assessing health-related characteristics of the built environment in a Brazilian city, and investigates whether agreement differs according to neighborhood income levels.

## Methods

We conducted an ecological study in São Leopoldo, a city in southern Brazil with a population of 214,087 inhabitants ^17^, using secondary data derived from a larger women population-based study. Sampling consisted of obtaining a representative sample of adult women from São Leopoldo (ages 20-69) in two stages: first, 45 census tracts were chosen by systematic random sampling from a list of 371 sectors ^17^ ordered by household income; then, 36 homes were selected from each tract ^18^. The unit of analysis for the present study was drawn from the locations of the homes in the 45 randomly selected census tracts. Based on the coordinates of the homes collected with a GPS (geographic position system) device (Etrex/Hcx/Garmin; https://www.garmin.com), we created midpoints for each tract using ArcGIS (http://www.esri.com/software/arcgis/index.html) around which 400m Euclidean buffers were generated to delineate neighborhoods ^18^. Since the generated neighborhoods covered a larger area than the census tracts, they encompassed almost all municipality regions and, according to the sampling design, represented different socioeconomic areas.

### Audit

We conducted an audit of the built environment for physical activity and dietary intake across the 45 neighborhoods. Using a map, pairs of auditors walked through 7 or 8 neighborhoods recording establishment information on a form, and noting the name, address, category, and GPS coordinates. Establishments were categorized into five groups: commercial establishments and services, food retail establishments for home consumption, food retail establishments for immediate consumption, physical activity establishments, and public leisure spaces. Commercial establishments and services included all types of stores and services available to the population, except pharmacies and health services. Food retail establishments for home consumption included supermarkets, grocery stores (neighborhood markets primarily selling food), convenience stores, bakeries, fruit shops, candy shops, and butcher shops. Food retail establishments for immediate consumption encompassed establishments that sell meals, snacks, and/or drinks for on-site consumption. The last two categories refer to spaces or establishments that offer leisure physical activities for the population, and were divided into physical activity establishments (gyms, sports schools, and health centers) and public leisure spaces (parks, squares, and open-air public recreation spaces). The audit was conducted on weekdays and Saturdays over a 5-month period (September 2015 to January 2016). A second evaluator reassessed 20% of the neighborhoods to test reliability, with most categories showing good or excellent agreement (≥ 0.6) and intraclass correlation coefficient (ICC) values ranging from 0.5 to 0.96. Audit data were exported to ArcGIS and all points outside the buffers were removed, with one neighborhood excluded due to missing data as it belonged to a neighboring municipality.

### Secondary data

Secondary data were obtained from lists of commercial establishments, services, and public leisure spaces provided by the city council. Data referring to commercial and food establishments, as well as services, were provided only for those in operation during 2015. Data on public leisure spaces consisted of one map with a list and localization of each space drawn in 2015. Establishment addresses were converted into geographic coordinates using Google Maps (https://maps.google.com). Establishments were categorized based on their name. Geographic coordinates were inputted into the open-source software DNRGPS (https://gisdata.mn.gov/dataset/dnrgps) and exported to ArcGIS to determine the number of establishments in each category for each neighborhood.

### Data analysis

Continuous variables (counts) and their distribution across the neighborhoods were analyzed using central tendency (means) and dispersion (standard deviation - SD) measures. Agreement between the audit and secondary data was estimated using ICC, adopting a 95% confidence interval (95%CI) and the following thresholds: excellent (ICC ≥ 0.75), good (ICC = 0.60-0.74), weak (ICC = 0.40-0.59), and poor (ICC < 0.40) ^19^. Analysis was performed for the entire area (all neighborhoods) and according to income terciles (mean income per capita: first tercile ≤ BRL 620.50; second and third terciles ≥ BRL 620.60), with neighborhoods in the first tercile having the lowest income per capita. Neighborhood income was defined using the mean income per capita in the census tract from which the centroids comprise the buffer ^17^. All analyses were performed using SPSS version 18.03 (https://www.ibm.com/).

## Results

**Figure 1:**
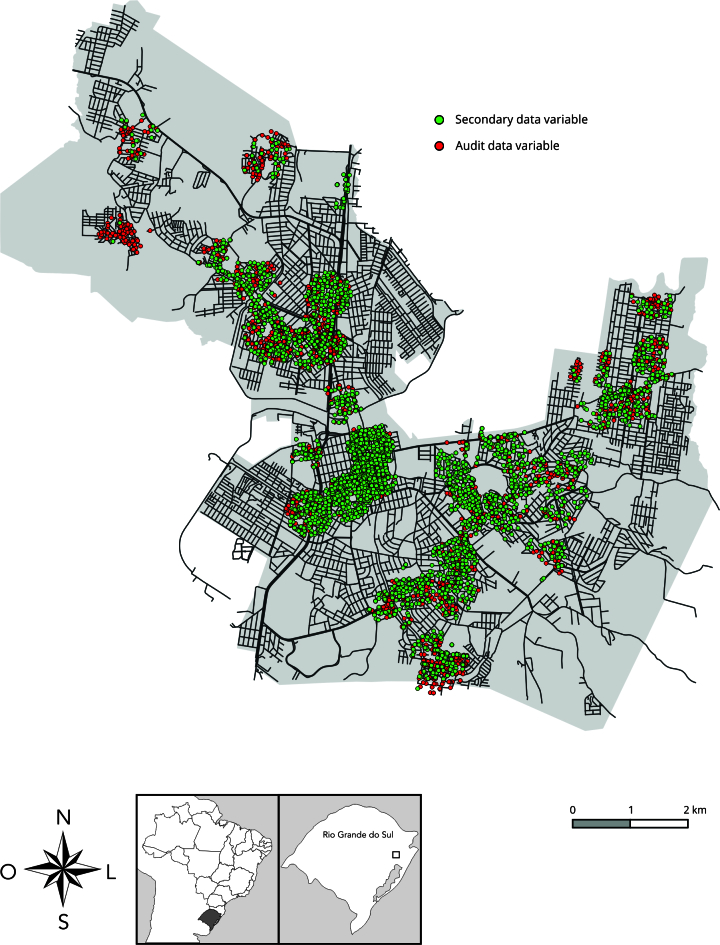
Distribution of audit and secondary data for built environment variables in São Leopoldo neighborhoods, Rio Grande do Sul State, Brazil, 2015.

Figure 1 illustrates the distribution of audit and secondary data for built environment variables in São Leopoldo neighborhoods. Comparison of the audit and secondary data for the total area revealed good or excellent agreement (ICC from 0.66 to 0.92) for all categories except food retail establishments for immediate consumption, for which agreement was weak (ICC = 0.54) (Table 1). Comparison according to neighborhood income revealed poor agreement for the categories “food retail establishments for home consumption”, “food retail establishments for immediate consumption”, and “physical activity establishments” in the first income tercile. Although this result was not statistically significant, ICC values for these categories were higher in the second and third terciles (0.81, 0.58 and 0.65, respectively). For public leisure spaces, agreement was excellent across all income terciles, whereas commercial establishments and services had good agreement (0.74) in the first tercile and excellent (0.92) in the second and third terciles (Table 1).

**Table 1:** Comparison of audit and secondary data for built environment variables in São Leopoldo neighborhoods, Rio Grande do Sul State, Brazil, 2015 (n = 44).

Variables	Audit		Secondary data		Neighborhood income *		
					Total	1st tercile (n = 15)	2nd and 3rd terciles (n = 29)
	Mean (SD)	Minimum-Maximum	Mean (SD)	Minimum-Maximum	ICC (95%CI)	ICC (95%CI)	ICC (95%CI)
Commercial establishments and services	97.3 (129.3)	11.0-705.0	133.4 (195.4)	11.0-726.0	0.92 (0.85; 0.96)	0.74 (0.20; 0.92)	0.92 (0.69; 0.97)
Food retail establishments for home consumption	9.0 (5.9)	0.0-30.0	9.0 (7.6)	0.0-32.0	0.65 (0.35; 0.81)	0.26 (-0.40; 0.70)	0.81 (0.53; 0.92)
Food retail establishments for immediate consumption	12.0 (15.9)	1.0-30.0	2.3 (4.7)	0.0-23.0	0.54 (0.10; 0.80)	0.06 (-0.25; 0.46)	0.58 (0.43; 0.81)
Physical activity establishments	1.4 (3.0)	0.0-14.0	0.7 (1.3)	0.0-6.0	0.66 (0.39; 0.82)	0.19 (-1.10; 0.70)	0.65 (0.27; 0.83)
Public leisure spaces	0.9 (0.8)	0.0-3.0	1.1 (0.9)	0.0-4.0	0.80 (0.59; 0.90)	0.89 (0.67; 0.96)	0.83 (0.58; 0.93)

95%CI: 95% confidence interval; ICC: intraclass correlation coefficien; SD: standard deviation.

Note: values in bold are statistically significant.

* Neighborhood income: 1st tercile (≤ BRL 620.50); 2nd and 3rd terciles (≥ BRL 620.60).

## Discussion

Results show that agreement between the audit and secondary data was good or excellent for most of the dietary intake and physical activity environment categories across the 45 neighborhoods. However, the analysis by income terciles showed poor agreement for three categories (food retail establishments for home consumption, food retail establishments for immediate consumption, and physical activity establishments) in low-income neighborhoods. Higher agreement was found in the other income terciles. For the categories “commercial establishments and services” and “public leisure spaces” agreement was at least good across all income terciles.

Excluding neighborhood income, we obtained good agreement between the two methods for almost all environmental categories, excepting food retail establishments for immediate consumption which showed weak agreement. This may be due to the high turnover rate for openings and closures for this type of establishment ^20^. Our analysis showed an underestimation of the number of food retail establishments for immediate consumption by secondary data. As government secondary data are updated annually, the audit was possibly better capable of capturing the temporal variation of such establishments. We also observed a variance in the concordance between secondary data and audit results according to type of establishment. This variation has already been described in the literature ^20^ ^,^ ^21^ and reinforces the importance of this type of analysis, as it indicates which categories of the food and physical activity environment available in secondary data are more suitable for research use.

Our findings revealed low agreement between the audit and secondary data for three physical activity and food establishment categories in low-income neighborhoods, aligning with a study conducted in Chicago (United States) that found similar results for convenience stores and fast-food establishments in low-income tracts ^21^. Greater proportion of small-scale commerce in so-called poorer neighborhoods is a reality in middle- and low-income countries ^22^. In Brazil, neighborhoods in São Paulo city with lower educational attainment showed a higher number of local grocery stores, bars, and fast-food restaurants than those with higher educational success levels ^23^. As mentioned, this type of commerce is seasonal and more variable, changing throughout the day ^20^ ^,^ ^22^. Moreover, poorer regions have a higher probability of harboring unregistered businesses, which therefore do not appear on government lists ^22^.

Regarding physical activity establishments, the weak agreement between the methods in the lowest income tertile may due to our use of only one source of secondary data. National studies found in the literature have assessed the physical activity environment using data sources such as commercial lists in addition to those provided by the government ^24^ ^,^ ^25^. Thus, physical activity establishments in the lowest income tertile may present specific characteristics left uncaptured by government data, such as locations for individualized sports practices which may be conducted in one’s own home. Finally, we cannot rule out a possible classification error by the auditors for some of the establishments evaluated ^20^, particularly those more prevalent in the lowest income tertile. Despite extensive training offered to the auditors, we must consider whether the classification system used accurately reflects the local reality ^26^. Our findings suggest that using secondary data to assess the built environment for physical activity and dietary intake in São Leopoldo may generate biased results, as the agreement varied according to neighborhood income. Hence, some authors recommend testing the secondary data precision by socioeconomic status, race, ethnicity, and urbanicity ^21^.

One study limitation is that we failed to crosscheck each establishment found by the audit with the secondary data, focusing rather on criterion-related validity. This hindered identifying the causes of agreement variance, both among the establishment categories and neighborhood income terciles, which may be explained by the high turnover of non-registered establishments, geocoding inaccuracies in the secondary data, or identification and categorization errors during the audit ^10^ ^,^ ^20^. Nonetheless, entire neighborhoods (including all streets) were audited, all establishments were identified using a GPS device to maximize location accuracy, and audit reliability was confirmed. Moreover, we used comparative variables for both data sources (counts) which are simple and important spatial exposure availability measures for environment and health studies ^27^. We assessed built environment variables that are key to investigate both physical activity and dietary intake. Finally, neighborhood income was evaluated using the mean per capita income in the census tracts from which the centroids comprise the buffer. This method for estimating neighborhood income may be a limitation, but our study underline that variations in area income and its relation with methods for assessing the built environment need further investigation.

## Final considerations

The present study revealed that neighborhood income levels impacted agreement across three of the five categories, suggesting that caution should be taken when using secondary data to assess the impact of the built environment on physical activity and dietary intake. Our findings indicate that accurately evaluating the built environment in low-income areas is difficult; nonetheless, additional research is necessary to compare various secondary data sources and the audit approach in low- and middle-income countries to enhance the quality of built environment measurements and inform intervention policies. Addition, improving secondary data would require more rapid updates in government data systems to capture the time variation of establishments, and to implement a classification system for establishments that is useful for research purposes.
